# Fabrication, Structural Characterization and Uniaxial Tensile Properties of Novel Sintered Multi-Layer Wire Mesh Porous Plates

**DOI:** 10.3390/ma11010156

**Published:** 2018-01-17

**Authors:** Liuyang Duan, Zhaoyao Zhou, Bibo Yao

**Affiliations:** National Engineering Research Center of Near-Net-Shape Forming for Metallic Materials, School of Mechanical and Automotive Engineering, South China University of Technology, Guangzhou 510640, China; 201410100179@mail.scut.edu.cn (L.D.); yao.bibo@mail.scut.edu.cn (B.Y.)

**Keywords:** stainless steel, metal porous materials, pore size distribution, tensile behavior, tensile strength

## Abstract

There is an increasing interest in developing porous metals or metallic foams for functional and structural applications. The study of the physical and mechanical properties of porous metals is very important and helpful for their application. In this paper, a novel sintered multilayer wire mesh porous plate material (WMPPs) with a thickness of 0.5 mm–3 mm and a porosity of 10–35% was prepared by winding, pressing, rolling, and subsequently vacuum sintering them. The pore size and total size distribution in the as-prepared samples were investigated using the bubble point method. The uniaxial tensile behavior of the WMPPs was investigated in terms of the sintering temperature, porosity, wire diameter, and manufacturing technology. The deformation process and the failure mechanism under the tensile press was also discussed based on the appearance of the fractures (SEM figures). The results indicated that the pore size and total size distribution were closely related to the raw material used and the sintering temperature. For the WMPPs prepared by the wire mesh, the pore structures were inerratic and the vast majority of pore size was less than 10 μm. On the other hand, for the WMPPs that were prepared by wire mesh and powder, the pore structures were irregular and the pore size ranged from 0 μm–50 μm. The experimental data showed that the tensile strength of WMPPs is much higher than any other porous metals or metallic foams. Higher sintering temperatures led to coarser joints between wires and resulted in higher tensile strength. The sintering temperature decreased from 1330 °C to 1130 °C and the tensile strength decreased from 296 MPa to 164 MPa. Lower porosity means that there are more metallurgical joints and metallic frameworks resisting deformation per unit volume. Therefore, lower porosities exhibit higher tensile strength. An increase of porosity from 17.14% to 32.5% led to the decrease of the tensile strength by 90 MPa. The coarser wires led to a bigger contact area between the interconnecting wires, resulting in a stronger sintering neck that exhibited higher tensile strength. The wire diameter increased from 81 μm to 122 μm and the tensile strength increased from 296 MPa to 362 MPa. The fracture morphology showed that the wires experience necking deformation and ductile fracture.

## 1. Introduction

Porous metal materials not only possess the overall characteristics of metal, but also a series of special properties, such as a high specific strength, high specific stiffness, high damping capacity, high thermal insulation, and so on. Products, such as ultra-light structural components, damping devices, filtration or purification plants, heat-sinks, and cooling systems are produced by metal porous materials and play a significant role in environmental protection, aerospace and many other fields. In most case, the mechanical properties of materials decide their usability [1,2,3,4,5,6]. The mechanical properties of metal porous materials depend on the original metals, but the manufacturing process, porosity, and pore structure of the metal porous materials also exert a great influence on their mechanical properties. For examples, Fei Xu and his companions [7] investigated the tensile behaviors of sintering stainless steel powder porous materials and discovered that when the porosity is raised from 22.1% to 40%, the tensile strength decrease from 150 MPa to 45 MPa; when the sintering temperature decreased from 1200 °C to 1150 °C, the tensile strength decrease from 150 MPa to 125 MPa. Russian professor Kostornov investigated the tensile properties at room temperature and at high temperature for the sintering metal fiber porous materials [8]. The results indicated that elongation decreases with the increase in porosity and that the tensile strength at room temperature is much higher than the tensile strength at high temperature. Recently, foamed aluminum has received much attention and research due to its high porosity and light mass. The constitutive model, named the Gibson-Ashby model, accurately describes the structure and performance of the open-cell foamed aluminum, and this model is widely approved in international community [9]. The completed research shows that when the relative density of the foamed aluminum is 25%, the tensile strength ranges between 6.2 MPa–10.8 MPa, and the Young modulus ranges between 4.1 GPa–6.0 GPa. Thus, it is clear that the mechanical properties of foamed aluminum are strongly associated with its porosity. On the other hand, this research also verifies the applicability of the Gibson-Ashby model.

The sintered metal wire/fiber porous materials are produced by compaction and the subsequent vacuum solid-phase sintering of metal wires or metal fibers. A Japanese professor Chino, Yasumasa put forward a methodology that takes advantage of the magnetic field force to layer the short metal fiber directionally, and based on the directional short fiber, to fabricate metal fiber felt. Thus, the metal skeleton and pores showed some form of regularity [10]. Professor Guo He developed the entangled metallic wire material, which was fabricated by long steel wires [11,12,13]. The steel wire was preformed through wire distortion and then sintered in a vacuum furnace. The uniaxial tensile property and compressive property for this porous metal was also investigated by their team. Professor Zhaoyao Zhou and his team provided a new method to fabricate a type of porous metal, known as porous twisted wire material by using the short fiber [14]. The wire skeleton and the pore structure of this porous metal were completely random and the pore size distribution was also irregular. Their research showed that when the porosity of the porous metal was 46%, the tensile strength was 112.75 MPa, which is much higher than that of the sintering powder porous metal at the same porosity [7]. The sintered multi-layer stainless steel wire mesh porous plate (WMPPs) had been designed and fabricated in this paper and the structure characteristics WMPPs had been investigated. In addition, the tensile tests for the WMPPs had been performed to investigate the effects of sintering parameters, porosities and wire diameters on the tensile properties. The deformation under the tensile stress and the fracture mechanism also had been analyzed. 

## 2. Experimental Procedures

### 2.1. Preparations of Materials

**Figure 1:**
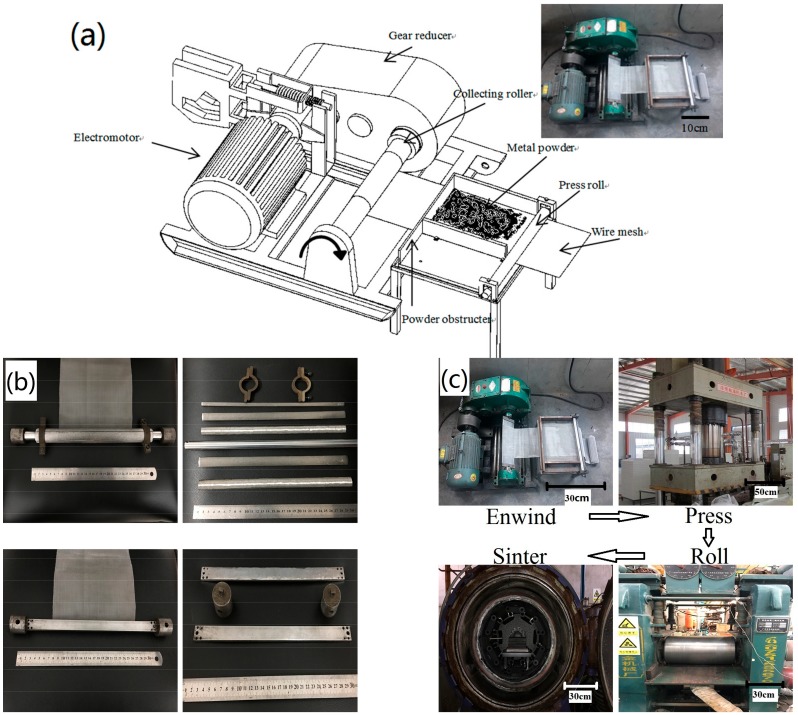
(**a**) The structure of the winding system; (**b**) the structure of the modularly designed core-plates; and (**c**) the fabrication process of the wire mesh porous plate (WMPPs).

According to different weaving techniques by warp and weft wires, there are three textile constructions: the plain-weave, the twill-weave and the stain-weave. The plain weave is the simplest and the constructions of the front and the back are all the same. In this paper, the plain-weave wire mesh of stainless steel was used to fabricate the sintered multilayer wire mesh porous plate (WMPP); stainless steel powder was also used. The fabrication method of the WMPP can be described in the following steps. First, the stainless steel wire mesh is fastened to a fixture, and the fixture is assembled in the collecting roller of an electric winch. There is a rectangular plate (or a cylinder), called the core-plate, which belongs to the fixture. Once the electric winch is pulsed-on, the wire mesh will be enwound on the plate tightly and rapidly. Before enwinding on the core-plate, the wire mesh goes through a platform, which is covered with metal powder. Above the platform by about 0.5 mm is a baffle plate, which ensures that the powder lying on the wire mesh is at a certain thickness. The metal powder will be enclosed in the interlamination of the multilayer wire mesh during the enwinding process on the core-plate. The enwinding process is shown in Figure 1a. After the enwinding process, a composite board that consists of the core-plate and the multilayer wire mesh with metal powder is obtained. Then, the core-plate is taken down from the composite board, and the remaining wire mesh and powder constitute the sample. The core-plate is of modular design for easy disassembly of the enwinding wire mesh and the core-plate, shown in Figure 1b. Next, the sample is pressed in a hydropress, and then the compacted sample is rolled in a rolling mill. In this way, the preform of the WMPP is obtained. Finally the sample is put into a vacuum furnace to sinter. The complete fabrication processes is shown in Figure 1c. 

### 2.2. Experimental Procedures

The pore structure and the pore skeleton were observed by an ultra-depth microscope (Keyence VHX-5000, KEYENCE, Osaka, Japan). A scanning electron microscope (Nova NANOSEM-430, Thermo Fisher Scientific Inc, Hillsboro, AK, America) was also used to observe the bonding state between the interlayers of the wire meshes and the appearance of fractures in the tensile samples. The tensile test was performed with an electronic universal mechanical testing machine (NO: AG-100NX, Shimadzu, Kyoto, Japan). 

The pore size and pore distribution are the primary parameters of the structural characteristics of the porous materials. In this paper, the pore size and pore distribution of the WMPPs were tested by use of the bubble point method. An Integrity Test Instrument (NO: GAOQ FLA-40, GaoQ Functional Materials Co., Ltd. Nanjing, China) was used to measure the pore size and the total size distribution. When the pores of porous materials are blocked by a wetting agent, a certain pressure is needed in order to open the pores. Because of the surface tension of the wetting agent and the small size of the pores, a high pressure is needed. In this experiment, the pores of the WMPPs were full of alcohol, and it was discharged by means of gas pressure. The relationship between gas pressure and pore size is formulated as:

$$D=\frac{4\gamma \cos\theta }{\Delta P}$$

where *D* is the pore diameter (μm), *γ* is the surface tension of wetting agent (N/m), *θ* is the wetting angle, Δ*P* is the differential pressure of the two sides of as-prepared samples (KPa).

The average porosities of sintered wire mesh porous plate (WMPP) are calculated by the quality-volume method, which is formulated as:

$$P\left(\%\right)=\left(1-\frac{M}{\rho V}\right)\times 100$$

where *P* is the average porosity of WMPP, *M* is the mass of WMPP (g), *V* is the volume of WMPP (cm^3^), and ρ is the density of the solid metal of original wire mesh (g/cm^3^).

## 3. Results and Discussion

### 3.1. Structural Characterization of the Sintered Multi-Layer Wire Mesh Porous Plates

**Figure 2:**
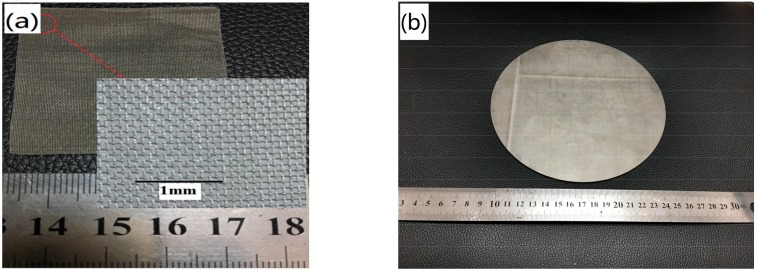

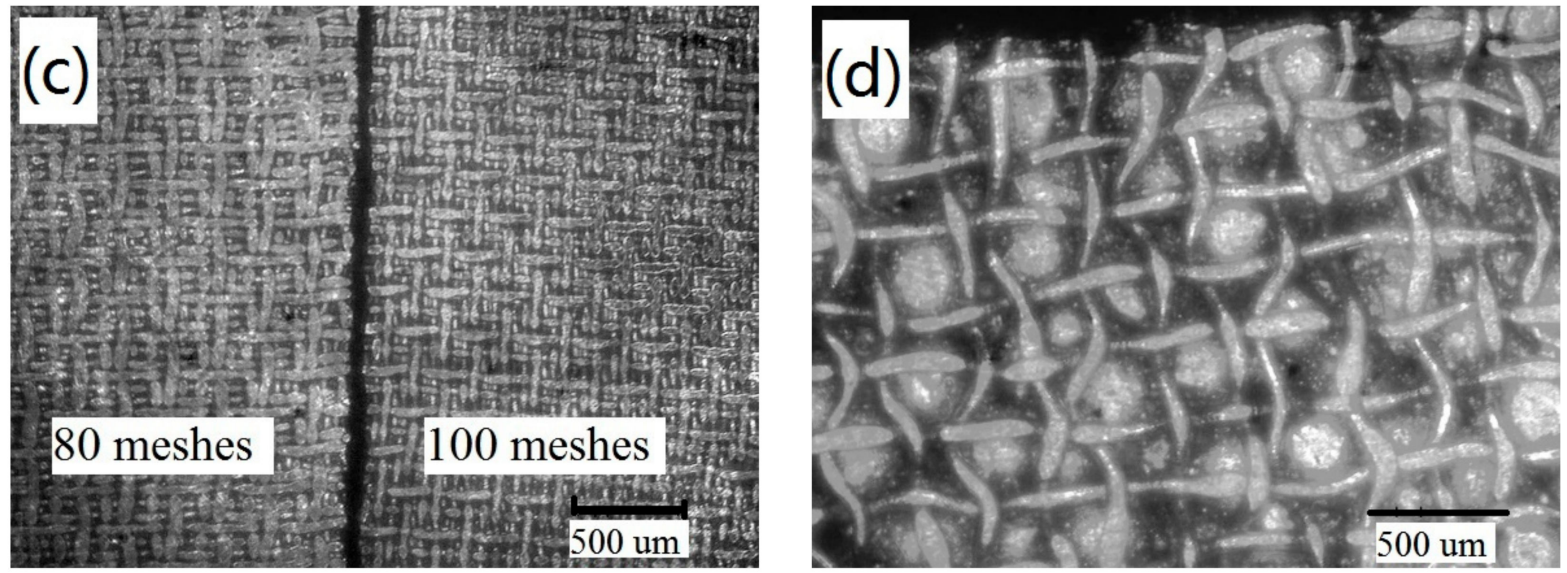
(**a**) The structure of the wire mesh with the 100 mesh-screen; (**b**) the appearance of the WMPPs; (**c**) the pore and skeletal structures of the WMPPs with different wire meshes; and, (**d**) the pore and skeletal structures of the WMPP made by wire mesh and powder.

Figure 2a shows the structure of the stainless steel wire mesh with 100 mesh-screens that are used for the fabrication of the WMPPs. From the enlarged view, we can see that the warp and weft wires of the stainless steel wire mesh are crisscrossed and form inerratic square meshes. The constructions of the front and back are identical. The wire diameter of the 100 mesh-screen was found to be 81 μm, with an aperture of 150 μm; the wire diameter of the 80 mesh-screen was found to be 100 μm, with an aperture of 180 μm; and, the wire diameter of 60 mesh-screen was found to be 122 μm, with an aperture of 220 μm.

Figure 2b shows the macro appearance of the WMPPs with a porosity of about 20%. The surface of the porous plate is smooth and flat, and the tiny pores are observed directly. The WMPP material exhibits very obvious porosity characteristics, and there is a distinct difference when compared to ordinary solid stainless steel plates. Figure 2c shows the enlarged view of the structure of the WMPPs with the 80 mesh-screen and 100 mesh-screen. The pores are regularly arranged and the pore shapes exhibit an inerratic square. Furthermore, the pore sizes are similarly and evenly distributed. The outermost layer of the wire mesh is pressed flat because it made direct contact with the roller during the rolling process, but there are no fractures. It is obvious that the wire diameter and pore size of the 80 mesh-screen are slightly bigger than those of the 100 mesh-screen. Figure 2d shows the structure of the WMPPs when prepared by use of the stainless steel powder enveloped in the 100 mesh-screen. From the image, we can see that the pores are distorted due to the extrusion force that is caused by the powder and that the shape of the pores is irregular, though the pore distribution is uniform.

**Figure 3:**
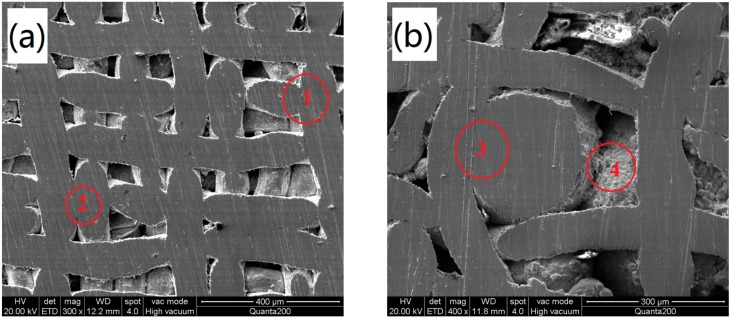
SEM images of the bonding types of WMPPs. (**a**) Sample made by wire mesh; and, (**b**) sample made by wire mesh and metal powder.

Figure 3 shows four primary types of sintering joints in the WMPPs. The first type of sintering joint is formed by the crisscross between the warp and weft wires of the stainless steel wire mesh, and they appear on single layer wire meshes or between adjacent layers of wire meshes. The second type of sintering joint is formed by the parallel warp or parallel weft wires of the wire mesh, and they appear only between adjacent layers of wire meshes. The above two sintering joints types are shown in Figure 3a. The third type of sintering joint is formed by the wires and powder contacting with each other. Obviously, the metal powder making contact with each other and sintering to bond together forms the fourth type of sintering joint. These two sintering joints types are shown in Figure 3b.

**Figure 4:**
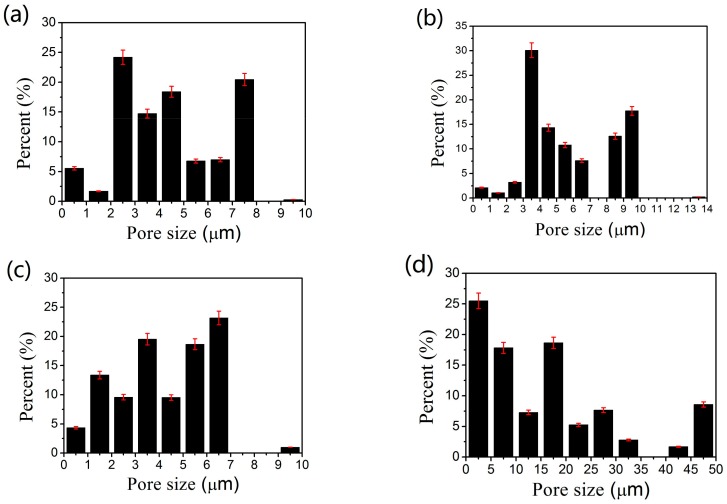
Pore size distribution of the sintered multilayer wire mesh porous plates. (**a**–**c**) the pore size distribution of the WMPPs which were prepared by mesh wire; and (**d**) the pore size distribution of WMPP which is prepared by mesh wire and metal powder.

Figure 4 shows the pore distribution of the as-prepared samples of WMPPs. For the sample prepared by the 100 mesh-screen, sintered at 1130 °C for 2 h (Figure 4a), the pore sizes show small scope changes and all of the pore diameters are less than 10 μm. For the sample prepared by the 80 mesh-screen (Figure 4b), the pore sizes also show small scope changes, with a few of the pore diameters exceeding 10 μm. However, in general, the pore sizes of the sample prepared by the 80 mesh-screen are bigger than the pore sizes prepared by the 100 mesh-screen. The data of the average pore size from Table 1 proves that the pore sizes of the WMPPs are dependent on the wire diameter and aperture of the original wire meshes. In Figure 4b, more than 30% of pores are 3 μm−4 μm, and the maximum size is 13.42 μm. The size distribution for this sample shows instability, perhaps hinting at the existence of uneven plastic deformation caused during the rolling process. For Figure 4c, the maximum size is 9.63 μm and the average size is 4.62 μm. Both of these values are smaller than the sample in Figure 4b. The porosity of sample c is 13.63%, and the porosity of sample b is 17.14%. This difference illustrates a larger rolling deformation, perhaps resulting in the smaller pore sizes. On the other hand, the sintering temperature for sample b and c are 1130 °C and 1330 °C, respectively. During the sintering process, the higher temperature signifies more intense atomic motion that leads to faster and larger sintering neck growth, which also reduces the pore size [15,16]. Figure 4d shows the pore distribution of a sample that was prepared by the powder enveloped in the 100 mesh-screen, and sintered at 1130 °C for 2 h. The pore size of this sample becomes larger and the size distribution becomes dispersive. A large span of sizes exists with an average size of 19.98 μm and a maximum size of 86.3 μm. During the rolling process, the powder should slide so that crevices (big pores) easily appear.

### 3.2. The Uniaxial Tensile Stress-Strain Behavior of the Sintered Wire Mesh Porous Plates

The tensile property is one of the most important mechanical performance indicators for structural materials. However, because of the large number of pores that exist in porous materials, the mechanical properties of porous materials are inferior to those of solid materials [17,18]. The tensile property for the WMPPs created from different processes is investigated in this paper.

**Figure 5:**
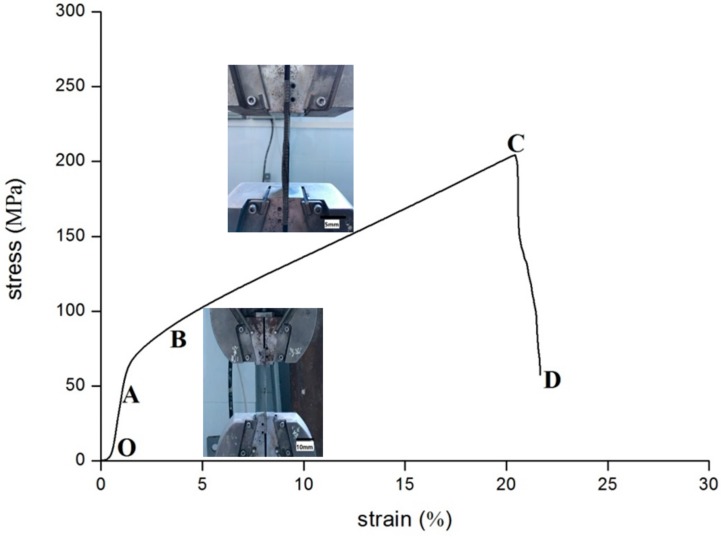
The typical tensile stress-strain curve for WMPPs.

The typical tensile stress-strain curve for WMPPs prepared by the 100 mesh-screen and sintered at 1230 °C for 2 h is presented in Figure 5. In Figure 5, the curve is divided into four stages: the initial elastic stage (O–A); the yielding stage where plastic deformation starts and the elasticity shows (A–B); further plastic deformation and the quick increase of stress reaching the maximum value (B–C); and, the appearance of the macro-crack and the stress level dropping sharply with the expansion of the crack leading to complete failure (C–D). According to the stress-strain curve, there is no obvious yielding point and the plastic deformation process in B–C increases slowly. On the other hand, the failure process in C–D decreases rapidly. The tensile curve exhibits short-term linear elastic deformation and the relationship between stress and strain conforms to Hook’s law. Owing to the short linear stage, the sample has little plastic deformation and the plastic strain is under 2%. The A–B stage is an arc, and the nonlinearity means that the plastic and elastic deformations occur concurrently. Somewhere, the local stress concentration leads the wires to reach the elastic limit first and start plastic deformation. Besides, the local yielding releases the local stress concentration and leads to stress redistribution, which results in the other wires starting elastic deformation [12]. Finally, all the wires reach the elastic limit and start plastic deformation. In the B–C stage, the deformation goes on and induces work-hardening, resulting in the gradual increase of stress, up to the maximum value. After point C, one or several wires have cracked and the stress redistribution causes the neighboring wires to crack as well. This cycle occurs rapidly until it reaches complete failure, which leads to a sharp drop in stress, as shown in the C–D stage. 

**Figure 6:**
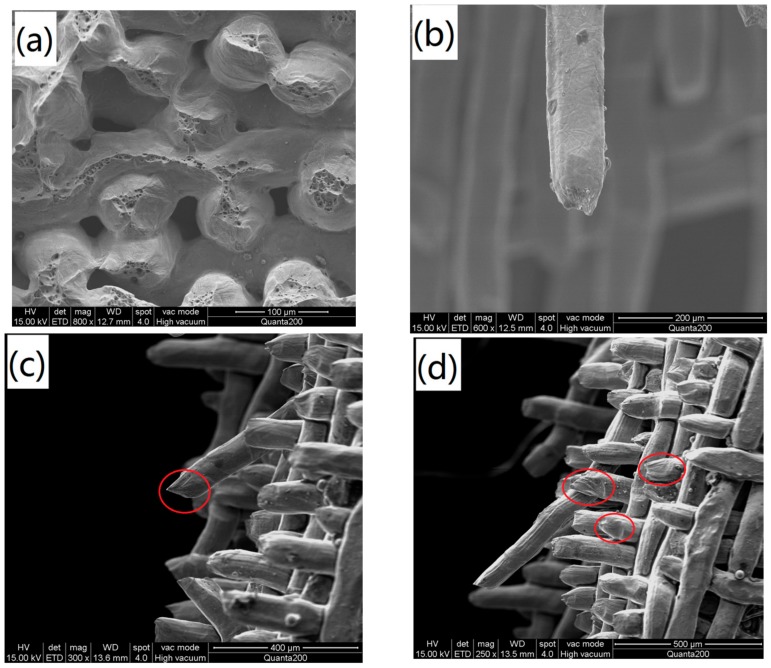
Fracture morphology (SEM) after the tensile test: (**a**) the end face with dimple; (**b**) the wire necking; (**c**) the wedge-shape fracture; and, (**d**) the tearing-mode fracture.

Figure 6 shows the fracture morphology after the tensile test of the WMPP sample. The figures explain the fracture process and the fracture mechanism of the tensile sample clearly. The bonding points inside the sample come from the cross points of the metal wires. In the rolling process, the cross points were subjected to a great deal of deformation and became vulnerable to tensile deformation. Thus, when the rupturing begins, the bonding points are broken (Figure 6a). From the fracture morphology of the wires, it is evident that they were subjected to plastic deformation and necking down (Figure 6b). The necking extent of the wires was small, which means that the wires were fractured rapidly without entirely going through plastic deformation. There are many dimples on the fracture, which explain that the wires went through ductile fracturing. Wedge-shape (Figure 6c) fractures and tearing-mode fractures (Figure 6d) also occurred. The fracture process can be described in the following manner. With the stretching going on, the entirety of the samples goes through elastic deformation and plastic deformation. As the deformation increases, the bonding points inside the sample are broken, and then the pores structures are loosed. With the structure loosing, the stress along the stretching direction is increased rapidly, leading to the cracks initiate and extend fast from the metallurgical defects, and resulting in the specimen rupture without complete plastic deformation [12]. This mechanism also gives the reason for the sharp drop in the stress-strain curve. 

**Figure 7:**
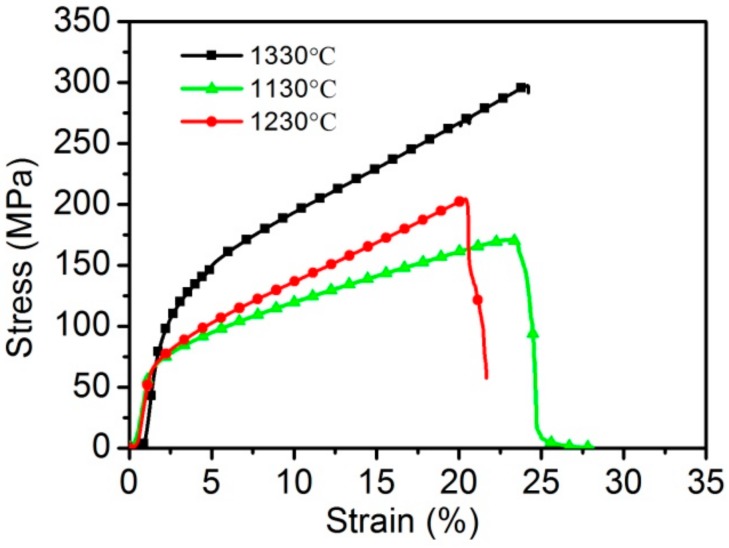
The uniaxial tensile stress-strain curves for the sintered wire mesh porous plates with different sintering temperatures.

Three different sintered multi-layer stainless steel wire mesh porous plates are fabricated and the sintering temperatures are 1130 °C, 1230 °C, and 1330 °C. The real porosities for the three samples are 18.27%, 18.3%, and 20%. The tensile stress-strain curves of the three samples are shown in Figure 7. The tensile properties of the three specimens which sintered at different temperature exhibit significant difference. The ultimate strengths for the samples sintered at 1130 °C, 1230 °C, and 1330 °C are 164 MPa, 204 MPa, and 296 MPa, respectively. Thus, it is clearly that the tensile strength rise along with the sintering temperature increasing. The tensile strength at 1330 °C is about 1.8 times of that at 1130 °C. When compared with the strength, the elongation at the ultimate stress and that of total failure don’t exhibit regular change along with the changing sintering temperature. The elongations at the ultimate stress are 24%, 20.5%, and 24.5%. The failure stage becomes faster with the increasing sintering temperature. 

After the rolling process, mechanical bonding occurs between the multilayer wire meshes. After the sintering process, the mechanical bonding changes into metallurgical bonding, resulting in great improvements in the bonding strength [19]. With the increase in the sintering temperature, the matter migration becomes quicker and leads to the growth and thickening of the sintering neck, which in turn, results in stronger sintering joints. On the other hand, the atom diffusion coefficient is larger at higher temperatures, perhaps resulting in the neighboring wires (no contact between the wires) achieving metallurgical bonding [20]. That means that more sintering joints occur at higher temperatures. The stronger sintering necks and more sintering joints can enhance the capacity of porous materials to resist deformation, leading to an increase in the ultimate strength with higher sintering temperatures. Therefore, the sintering temperature plays an important role in the structural effects and mechanical properties of WMPPs.

**Figure 8:**
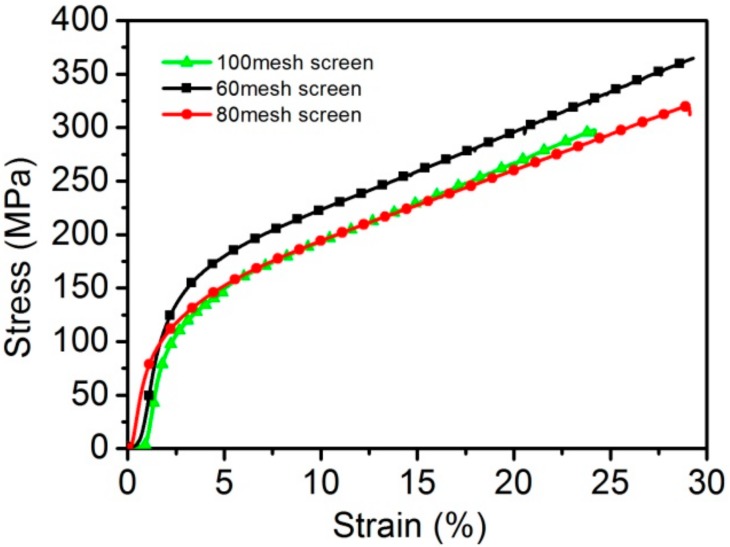
The uniaxial tensile stress-strain curves for the sintered wire mesh porous plates with different mesh-screens.

Figure 8 shows the tensile stress-strain curves of WMPPs prepared with the 60 mesh-screen, the 80 mesh-screen, and the 100 mesh-screen, with a sintering temperature of 1330 °C for 2 h. The real porosities of the three samples are 21.3%, 20.6%, and 20%, respectively. The wire diameters of these three wire meshes are shown in Table 1. The three stress-strain curves possess similar trends of variation. It is also worth noting that the failure stages of the three curves are very short, which means that the process from the crack initiation to the total failure occurs in an instantaneous. The fracture mechanism of these three samples is similar to that of solid stainless steel, and this phenomenon demonstrates that the mechanical properties of the WMPPs prepared at 1330 °C are close to that of solid stainless steel [21]. The tensile strengths of the three samples are different; the largest one is the sample prepared with the 60 mesh-screen with a value of 363 MPa, then comes the 80 mesh-screen sample with 324 MPa, and then finally the 100 mesh-screen sample with 296 MPa. In other words, the tensile strength increases with the thickness of the wire. The total failure elongations of these samples are 29.8%, 29.2%, and 24.8%, respectively. Because of the short failure process, the maximum stress elongation closely approximates to that of the total failure elongation.

These differences indicate that the mechanical properties of the WMPPs strongly depend on the diameters of the wires. Under pressure, we know that the contact area between two thin wires is smaller than the contact area between two thick wires. Because the separate wires get together by pressure and sintering, they would have a larger binding force if the contact areas were big between the wires. For this reason, the WMPPs made using thick wire are superior to porous metals made using thin wires in terms of tensile properties.

**Figure 9:**
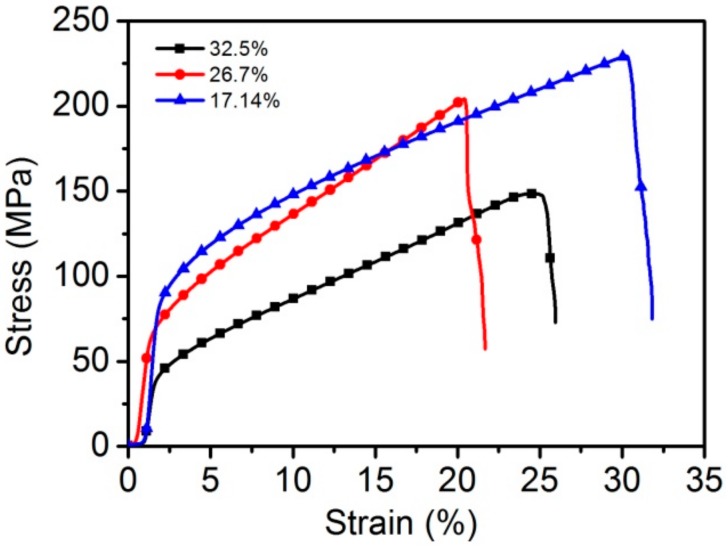
The uniaxial tensile stress-strain curves for the sintered wire mesh porous plates with different porosities.

Figure 9 shows the tensile stress-strain curves of the WMPPs with real porosities of 17.14%, 26.27%, and 32.5%. These samples are prepared with 80 mesh-screen and sintered at 1230 °C for 2 h. The tensile strengths of these samples are 230.6 MPa, 210 MPa, and 140.5 MPa, respectively. As with other porous materials, the mechanical properties of the WMPPs strongly depend on the porosity. The principle of the phenomenon is easy to explain. Lower porosity means that there are more wires per unit volume participating in the process of resisting tensile deformation, resulting in structures with lower porosity exhibiting higher tensile strength. On the other hand, lower porosity contains more metallurgical joints per unit volume, leading to the capacity of resistance against deformation to increase [7,12]. Therefore, the tensile strength increases with the decreasing porosity.

The summary of uniaxial tensile properties for the WMPPs is shown in Table 2. From the Ultimate Tensile Strength column, we can see that even the lowest value (140 MPa), which belonged to the sample with 32.5% porosity and sintered at 1230 °C for 2 h, is much higher than that of other porous materials, such as foamed aluminum, foamed nickel, sintered metal powder materials, and sintered metal fiber materials with the same porosity [4,5,7,8]. In my opinion, the advantageous performance of the WMPPs is due to the continuous wires (the pore skeletons) and the inerratic pore structure. 

## 4. Conclusions

The sintered multi-layer stainless steel wire mesh porous plates (WMPPs) are fabricated by enwinding, rolling, and the subsequent vacuum sintering. The pore structures of the WMPPs prepared with a single wire mesh are regular, and there is no curve or torsion for the wires. Furthermore, the pore shapes are rectangular. The pore structures of the WMPPs prepared with the wire mesh and metal powder are distorted due to the extrusion force caused by the powder, and the shapes of the pores are irregular.

The average pore diameters of the WMPPs with porosity of 18% prepared by 100 mesh screen and 80 mesh screen are 4.16 μm and 5.5 μm, respectively, which indicate that the wire diameters and pore sizes of original wire mesh have some influence on the pore size of WMPPs. The average pore diameters of the WMPPs with the porosities of 13.63% and 17.14% prepared by 100 mesh screen are 5.5 μm and 4.62 μm, respectively, which indicate that lower porosity leads to smaller pores. The average pore diameter of the specimen with 29.1% porosity prepared by wire mesh and metal powder is 19.98 μm and the maximum pore is 86.3 μm. The pore size for this sample has a wide distribution. 

According to the results of the tensile experiences, the sintering temperature has a large effect on the tensile properties of WMPPs. The ultimate strength for the WMPPs with porosity of 20% increases from 164 MPa to 204 MPa, with an increasing sintering temperature from 1130 °C to 1330 °C, which clearly shows that the tensile strength rises along with the increasing sintering temperature.

The porosity of the WMPPs has a clear effect on their tensile properties. According to the experiment data, with the porosity increased from 17.1% to 32.5%, the tensile strength decreased from 230.6 MPa to 140.5 MPa, which indicates that the tensile strengths of the WMPPs decrease with the increasing porosity. 

The tensile behavior strongly depends on the wire diameters. The specimen prepared with 60 mesh-screen exhibits the ultimate strength of 363 MPa. However, the specimen prepared with 100 mesh-screen exhibits the ultimate strength of 296 MPa. Thus, the tensile strength of the WMPPs with coarse wire is higher than that of WMPPs with fine wire.

## Figures and Tables

**Table 1:** Summary of porous size of the sintered wire mesh porous plates.

No.	Mesh Number	Sintering Parameters	Porosity (%)	Maximum Pore Size (μm)	Average Pore Size (μm)	Pore Size Distribution (μm)
a	100	1130 °C × 2 h	18.3	9.9	4.1	0.2~9.9
b	80	1130 °C × 2 h	17.1	13.4	5.5	0.3~13.4
c	80	1330 °C × 2 h	13.6	9.6	4.6	0.2~9.6
d	Mesh and powder	1130 °C × 2 h	29.1	86.3	19.9	2.9~86.3

**Table 2:** Summary of the uniaxial tensile properties for the sintered wire mesh porous plates.

Mesh Number	Sintering Parameters	Porosity after Sintering (%)	Ultimate Tensile Strength (MPa)	Elongation at Total Failure (%)
100	1330 °C × 2 h	20.0	296	24.8
100	1230 °C × 2 h	18.3	204	22.5
100	1130 °C × 2 h	18.3	164	28.0
80	1330 °C × 2 h	20.6	324	29.6
80	1230 °C × 2 h	17.1	230	32.0
80	1230 °C × 2 h	26.2	210	22.0
80	1230 °C × 2 h	32.5	140	26.0
60	1330 °C × 2 h	21.3	363	29.8
